# A mariner transposon vector adapted for mutagenesis in oral streptococci

**DOI:** 10.1002/mbo3.171

**Published:** 2014-04-21

**Authors:** Martin Nilsson, Natalia Christiansen, Niels Høiby, Svante Twetman, Michael Givskov, Tim Tolker-Nielsen

**Affiliations:** 1 Costerton Biofilm Center, Department of International Health, Immunology and Microbiology, Faculty of Health and Medical Sciences, University of CopenhagenCopenhagen, Denmark; 2 Department of Clinical Microbiology, University Hospital, RigshospitaletCopenhagen, Denmark; 3 Department of Odontology, Faculty of Health and Medical Sciences, University of CopenhagenCopenhagen, Denmark; 4 Singapore Centre on Environmental Life Sciences Engineering, SCELCE, Nanyang Technological UniversitySingapore

**Keywords:** Biofilm, extracellular matrix, glycosyltransferases, mariner, mutagenesis, *Streptococcus mutans*, transposon.

## Abstract

This article describes the construction and characterization of a mariner-based transposon vector designed for use in oral streptococci, but with a potential use in other Gram-positive bacteria. The new transposon vector, termed pMN100, contains the temperature-sensitive origin of replication *repATs*-pWV01, a selectable kanamycin resistance gene, a *Himar1* transposase gene regulated by a xylose-inducible promoter, and an erythromycin resistance gene flanked by himar inverted repeats. The pMN100 plasmid was transformed into *Streptococcus mutans* UA159 and transposon mutagenesis was performed via a protocol established to perform high numbers of separate transpositions despite a low frequency of transposition. The distribution of transposon inserts in 30 randomly picked mutants suggested that mariner transposon mutagenesis is unbiased in *S. mutans*. A generated transposon mutant library containing 5000 mutants was used in a screen to identify genes involved in the production of sucrose-dependent extracellular matrix components. Mutants with transposon inserts in genes encoding glycosyltransferases and the competence-related secretory locus were predominantly found in this screen.

## Introduction

Oral streptococci are Gram-positive, facultative anaerobes that prevail on tooth surfaces in complex multi-species biofilms called dental plaque. Occasionally, due to environmental factors, the biofilm becomes cariogenic. There is a strong correlation between a prolonged acidic environment at the enamel and an increased population of *Streptococcus mutans*, which in the end leads to an accelerated progress of caries (Takahashi and Nyvad 2008). *S. mutans* has developed a variety of mechanisms to colonize the tooth surfaces and to outnumber other bacteria in a cariogenic biofilm (Lemos et al. 2005; Ahn et al. 2006). Oral streptococci may also enter the blood stream, not only through dental procedures but also during daily activities like eating and tooth brushing. In the blood circulation, they can potentially bind to for example laminin, fibrin, collagen, and platelets, and subsequently adhere to damaged heart valves and cause endocarditis (Beg et al. 2002; Sato et al. 2004).

Mutant library screens have provided significant data with regard to identification of genes responsible for specific phenotypes in bacteria. A number of different transposon-based gene delivery systems have been used for creating mutant libraries in bacteria. In the case of oral streptococci, transposon mutagenesis has been carried out with Tn916, Tn4001, Tn917 and ISS1 (Spatafora et al. 1995; Gutierrez et al. 1996; Boyd et al. 2000a,b). Tn916 harbors a preferred insertion site consisting of an A-rich sequence separated by six bases from a T-rich sequence. Tn10 needs a 6-bp unique target sequence, and Tn5 is dependent on host factors and not adapted to some bacteria (Choi and Jung, 2009). The Tn917 transposon has been shown not to be inserted randomly in the chromosome of various bacteria (Slater et al. 2003; Shi et al. 2009). Moreover, transposon mutagenesis with ISS1 may be problematic as some *Streptococcus* species contains endogenous ISS1 elements that can recombine with the inserted ISS1, resulting in chromosomal deletions (Thibessard et al. 2002). We sought to develop a system for unbiased transposon mutagenesis in oral streptococci. To this end we employed the mariner transposon, which has been shown to insert at random positions in the bacterial chromosomes that have been analyzed so far, and which does not have endogenous counterparts in bacteria (Lampe 2010). In addition, the recognition signal for insertion of the mariner transposon is only two basepairs (TA dinucleotides) (Lampe et al. 1999). Among streptococci, the mariner system was first used in *Streptococcus pneumonia* using an in vitro mutagenesis protocol, and later developed as a signature-tagged mutagenesis system in *Streptococcus sanguinis* (Akerley et al. 1998; Paik et al. 2005).

The plasmid pBTn contains a xylose-inducible *Himar1*-transposase gene that mediates transposition of an erythromycin resistance gene flanked with himar inverted repeats (Li et al. 2009). pBTn contains a temperature-sensitive origin of replication that is functional in *Staphylococcus aureus* and *Bacillus subtilis*, but not in streptococci. The plasmid pTV1-OK contains genes required for transposon Tn917 mutagenesis (Gutierrez et al. 1996), as well as the temperature-sensitive origin of replication *repATs*-pWV01 which is functional in streptococci. In this study, we have fused parts of the pBTn and pTV1-OK plasmids to obtain a new plasmid, pMN100, which contains the temperature-sensitive origin of replication *repATs*-pWV01, the selectable kanamycin resistance gene *aphA3*, the *Himar1* transposase gene regulated by a xylose-inducible promoter, and an erythromycin resistance gene flanked by himar inverted repeats. We transformed *S. mutans* UA159 with pMN100 and have provided evidence that transpositions occur with an unbiased distribution in the genome. In addition, as a proof of concept, we created a mariner transposon mutant library containing 5000 mutants, and performed a screen to identify genes involved in the production of sucrose-dependent extracellular matrix components.

## Materials and Methods

### Bacterial strains and growth conditions

The bacterial strains and plasmids used in this study are listed in Table 1. For routine cultivation *S. mutans* UA159 (Ajdic et al. 2002), belonging to Bratthall serotype c, was grown in Tryptone soya broth (TSB) at 37°C aerobically. Tryptone soya agar (TSA) with and without sucrose were used for plating and anaerobic incubation (10% H_2_, 10% CO_2_ and 80% N_2_). *Escherichia coli* strains were grown in Luria-Bertani medium at 37°C. Where appropriate, antibiotics were used for bacterial cultures at the following concentrations (if nothing else is stated): for *S. mutans* strains, erythromycin (Sigma-Aldrich, St. Louis, MO) at 5 *μ*g/mL, kanamycin (AppliChem GmbH, Darmstadt, Germany) at 300 *μ*g/mL; for *E. coli* strains, kanamycin at 50 *μ*g/mL.

**Table 1:** Strains and plasmids used in this study

Strain or plasmid	Relevant characteristics or sequence	Source orreference
*Streptococcus mutans*		
UA159	American type culture collection (ATCC 700610)	Ajdic et al. (2002)
*E. coli*		
HB101	*recA thi pro leu hsdRM*, Sm^r^; strain used for maintenance and proliferation of plasmids	Kessler et al. (1992)
DH5*α*	F^−^, *ϕ*80d*lacZ*ΔM15, Δ(*lacZYA-argF*)U169, *deoR, recA*1, *endA*1, *hsdR*17(rk^−^, mk^+^), *phoA, supE*44, *λ*^−^, *thi*-1, *gyrA*96, *relA*1	Invitrogen
Plasmids		
pBTn	1.45 kbp ermB fragment of Tn551, xylose-inducible promoter and *xylR, Himar1* transpose gene	Li et al. (2009)
pTV1-OK	*repATs*-pWV01Ts *aphA3* Tn917(*erm*)	Gutierrez et al. (1996)
pMN100	*repATs*-pWV01Ts *aphA3* 1.45 kbp ermB fragment of Tn551, xylose-inducible promoter and *xylR, Himar1* transpose gene	This study

### Construction of the pMN100 transposon vector

**Figure 1:**
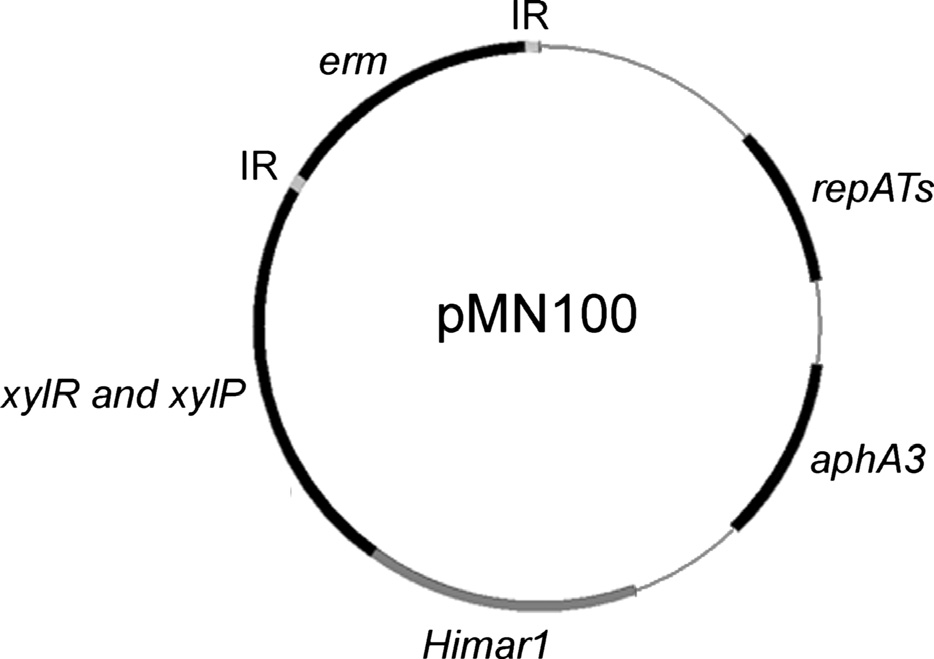
Schematic of the *Himar1*-based transposon vector pMN100. Gene designations: *repATs*-pWVO1, temperature-sensitive origin of replication; *aphA3*, kanamycin resistance gene; *erm*, erythromycin resistance gene; *xylR* and *xylP*, xylose regulator gene and promoter; *Himar1*, transposase gene; IR, mariner inverted repeats.

The pMN100 transposon vector (see Fig. 1) was constructed as follows. Purified pTV1-OK (Oiaprep® Spin Miniprep kit; Qiagen GmbH, Hilden, Germany) was digested with *EcoRI* and *PstI*. The 3.5 kb backbone fragment, containing the temperature-sensitive *repATs* replicon and the *aphA3* gene encoding kanamycin resistance, was agarose gel extracted, blunt ended with T4 DNA polymerase and dephosphorylated with shrimp alkaline phosphatase. pBTn was digested with *KpnI* and *PstI,* and the 4.4 kb fragment, containing the *Himar1* transposase gene, the xylose-inducible promoter, the xylR regulator gene and the erythromycin resistance gene flanked by himar inverted repeats, was agarose purified and blunt ended as described above. The fragments were ligated and transformed into *E. coli* HB101 (Kessler et al. 1992). The new transposon vector, named pMN100, was confirmed by restriction analyses and partial sequencing.

### Transformation of *S. mutans*

The transformation method was based on previously published procedures (Cvitkovitch et al. 1998). A volume of 250 *μ*L from an *S. mutans* overnight culture grown in Todd-Hewitt medium (THB) was added to 12 mL THB containing 25% heat-inactivated horse serum (PAA Laboratories GmbH, Pasching, Austria). The cultures were incubated at 37°C anaerobically, and when they reached an OD_600_ between 0.15 and 0.25 they were left at room temperature for 10 min. The pMN100 plasmid was added (∼1 *μ*g) and the tubes were incubated at 30°C for 1 h. Cultures were diluted with 5 mL THB supplemented with horse serum and incubated for an additional hour. Cells were harvested by centrifugation and, resuspended in THB and spread on TSA plates containing erythromycin and 2 mg/mL kanamycin. The plates were incubated at 30°C for 3 days.

### Transposon mutagenesis in *S. mutans*, and mutant library preparation

An overnight culture of *S. mutans*/pMN100, grown at 30°C in TSB supplemented with kanamycin and erythromycin, was diluted 1:100 into tubes containing 1 mL TSB (without dextrose) supplemented with 0.5% xylose and kanamycin and erythromycin. The cultures (usually 300) were incubated for 24 h at 30°C. Ten *μ*L from each culture was subsequently transferred to tubes containing 1 mL TSB supplemented with erythromycin and incubated at the restrictive temperature of 40°C for 24 h. Then, 100 *μ*L of each culture was plated on TSA plates containing 10 *μ*g/mL erythromycin, and the plates were incubated at 37°C for 48–72 h. Five colonies per transposition culture were picked separately to a kanamycin and an erythromycin agar plate, and the plates were incubated for 48 h. Generally, three clones per transposition culture, with an Erm^r^ and Km^s^ phenotype, were picked to microtiter plates containing 100 *μ*L of TSB and grown for 24 h at 37°C. Glycerol was added to an end concentration of 15% and the plates were preserved at −80°C for future screening. All cultivations, except for the start overnight culture, were done in 2 mL eppendorf tubes. To create a library of 5000 clones, ∼1700 separate transposition cultures were grown.

### Identification of interrupted genes by arbitrary primed PCR

In order to identify the location of the mariner transposon insertions, the arbitrary primed PCR protocol outlined by Li et al. (2009) was followed. Briefly, in the first round of PCR one of the arbitrary primers was paired with a primer that reads out from the erythromycin resistance gene. Approximately 100 ng of purified chromosomal DNA, obtained using Qiagen's DNeasy® Blood & Tissue kit, was used as template. In the second PCR a part of the product from the first PCR was PCR amplified with an arb-3 primer and a nested *erm* primer. Purified second-round products were used for DNA sequence analyses performed by Macrogen by the use of the nested *erm* primer. Insertion sites were identified with BLASTN searches, against the annotated sequence of *S. mutans* UA159, using software provided by NCBI.

### Southern blot analysis

Southern blot analysis was performed with a DIG labeling kit and NBT/BCIP tablets were used for detection according to the manufacturer's (Roche Diagnostics GmbH, Mannheim, Germany) protocols. The amplified 1.3 kb product using primer IRXbaI and IRPstI and comprising the erythromycin gene was used as a probe (Li et al. 2009). Chromosomal DNA obtained from mutants was digested with *EcoRI*.

### Colony morphology assay

Primary screening was made by plating spots directly from transposon library microtiter plate glycerol stocks onto TSA plates (14 cm diameter petri dishes) containing 1% sucrose, using a replicator with 3-mm pins. The plates were incubated at 37°C for 2 days. The colony morphology of collected semi-rough colony morphology mutants from the primary screening was confirmed in a second assay where 5 *μ*L from newly made glycerol stocks were spot-inoculated on TSA with and without 1% sucrose. The plates were incubated at 37°C for 3 days. Pictures of colonies from the second plate assay were taken with a Canon EOS 400D camera (Canon Inc.,Tokyo, Japan).

## Results

### Construction of a mariner transposon vector suitable for use in oral streptococci

A mariner transposon vector was constructed with the aim to perform unbiased transposon mutagenesis in oral streptococci. The components of the new transposon vector were taken from the plasmids pTV1-OK and pBTn (Gutierrez et al. 1996; Li et al. 2009). The backbone of pTV1-OK, containing the temperature-sensitive origin of replication *repATs-*pWV01 and the selectable kanamycin resistance gene *aphA3*, was fused with the pBTn part comprising an erythromycin gene flanked with himar inverted repeats and a *Himar1* transposase gene regulated by a xylose-inducible promoter. The new transposon vector, named pMN100, is schematically shown in Figure 1. The pMN100 plasmid can replicate in streptococci grown at 30°C. Expression of the *Himar1* transposase can be induced with xylose, and it promotes transposition of the erythromycin resistance gene flanked with himar inverted repeats. The pMN100 plasmid cannot replicate at 40°C and the bacteria will, therefore, be cured from the plasmid if they are grown at 40°C. Bacteria in which the erythromycin resistance gene is inserted into the chromosome will display erythromycin resistance at 40°C.

### Establishment of a mariner transposition protocol for *S. mutans*

The mariner transposon vector pMN100 was introduced into *S. mutans* UA159. Restriction analysis of plasmids purified from *S. mutans*/pMN100 transformants grown at permissive temperature indicated that the plasmid is structurally stable in *S. mutans*. The ability of the *S. mutans*/pMN100 strain to mediate transposition was investigated, using basically the transposition protocol described by Li et al. (2009) for pBTn-mediated transposition in *S. aureus*. The procedure resulted in integration of the erythromycin gene at TA dinucleotides in the chromosome of *S. mutans* UA159 (data not shown), confirming that the pMN100 plasmid functions as a transposon vector as anticipated. However, the procedure was not optimal since the number of unique clones per transposition culture was low (data not shown).

In order to be able to create comprehensive transposon libraries, we set up a novel transposition protocol. In the protocol described by Li et al. (2009) cultures were grown at permissive temperature, and were then cultivated for 24 h at restrictive temperature three times before plating. With *S. mutans* harboring pMN100, it was possible to obtain a high frequency (above 90%) of cured (i.e., plasmid-free) bacteria after only one overnight cultivation at restrictive temperature. This improvement, besides preparing transposition cultures in small volumes, that is, eppendorf tube scale, made it possible to perform a high number of transpositions in a short time. *S. mutans*/pMN100 was grown to stationary phase in TSB supplemented with erythromycin and kanamycin at permissive temperature, and the culture was then diluted into several tubes containing TSB (without dextrose) supplemented with xylose, erythromycin and kanamycin and these cultures were grown at permissive temperature overnight. The resulting stationary phase cultures were diluted into TSB containing erythromycin, and were then incubated overnight at restrictive temperature. Thereafter a part of these cultures were spread on TSA plates with erythromycin, and the plates were incubated at restrictive temperature. Colonies on the plates appeared after 2–3 days of incubation. Thereafter the putative transposon mutants were picked and analyzed separately on erythromycin and kanamycin plates. The frequency of temperature resistant Erm^r^ and Km^s^ clones, after a transposition procedure, was generally above 90%. When we applied our new transposition protocol, the sizes of the mutant colonies were more heterogeneous compared with the outcome when we followed the protocol described by Li et al. (2009). This was expected as our protocol involves only one cultivation at the restrictive temperature whereas the protocol of Li et al. (2009) contains three consecutive cultivations at the restrictive temperature. Throughout mutant library preparation we chose not to pick the smallest colonies in order to avoid mutants with slow growth. For library preparation, three Erm^r^ and Km^s^ clones were chosen per transposition culture, and thereby 60% of the mutants had unique inserts (i.e., were non siblings), as judged by DNA sequencing of 30 randomly picked mutants. Southern blot analysis of 10 mutants showed that only one transposon integration per mutant had occurred (data not shown), which is in agreement with the observed low frequency of transposon integration.

### Evidence for unbiased mariner transposon mutagenesis in *S. mutans*

**Figure 2:**
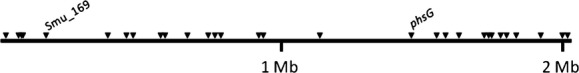
Mapping of unique mariner transposon insertion sites in 30 randomly picked *S. mutans*UA159 transposon mutants. The 2.03 Mbp genome of *S. mutans*UA159 is shown linearized. Arrow heads indicate approximate transposon insertion sites. Two gene names are included to indicate genome orientation.

Mariner tranposons have been shown to insert at random positions in the bacterial chromosomes that have been analyzed so far. To investigate if mariner transposon insertion occurs at random sites also in the *S. mutans* UA159 chromosome, the insertion sites in 30 randomly picked transposon mutants were determined by arbitrary primed PCR followed by sequencing of the PCR product. As shown in Figure 2, the transposon inserts are well distributed over the entire *S. mutans* UA159 genome, indicating that mariner transposon mutagenesis is unbiased in *S. mutants*. There are a few larger gaps, for example, between 1.14 and 1.46-Mbp, but this is likely attributed to the presence of clusters of essential genes.

### Isolation of mutants defective in the production of sucrose-induced extracellular matrix components

**Figure 3:**
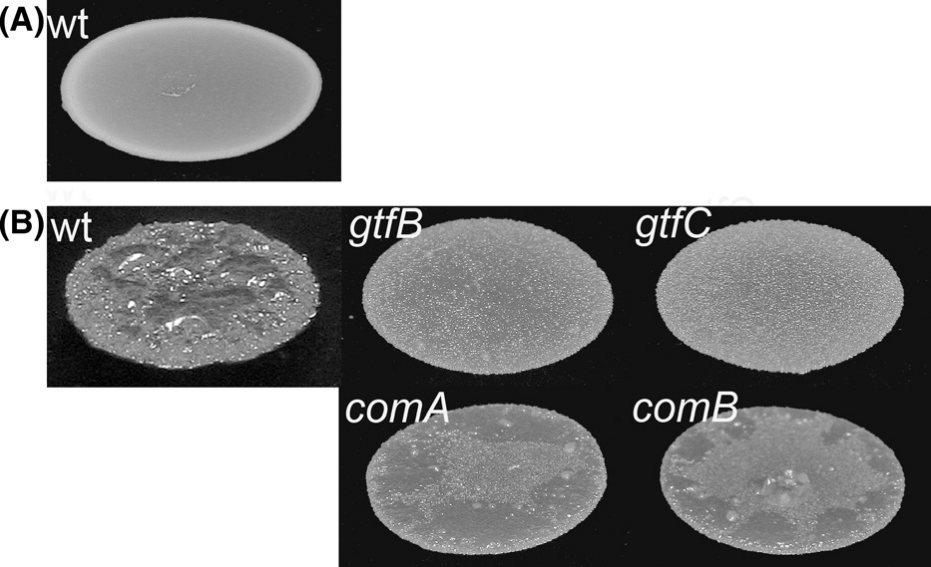
Colony morphology on TSA agar medium without sucrose (A) and with sucrose (B) of *S. mutans*UA159 wild type and the selected transposon mutants *gtfB, gtfC, comA,* and *comB*.

We created a *S. mutans* mariner transposon mutant library containing 5000 mutants, and performed a screen to identify genes involved in the production of sucrose-dependent extracellular matrix components in *S. mutans*. The formation by bacteria of wrinkled or rough colonies on solid medium is indicative of the production of extracellular matrix components such as exopolysaccharides, large adhesive proteins and aggregative fimbriae (Rainey and Travisano 1998; Friedman and Kolter 2004; Simm et al. 2004; Fazli et al. 2013). The fact that *S. mutans* forms rough colonies on solid medium containing sucrose, whereas it forms smooth colonies on solid medium without sucrose (Fig. 3) indicates that it produces sucrose-dependent extracellular matrix components. This provided a basis for screening of the mutant library for mutants that are defective in the production of sucrose-dependent extracellular matrix components. However, we were not able to isolate any mutant that gave rise to a smooth colony morphology on TSA sucrose plates similar to the colonies of the *S. mutans* wild type growing on TSA agar without sucrose. Instead, we isolated mutants with semi-rough colony morphology. The isolated mutants had a flatter colony structure with a more transparent appearance compared to the wild type colony morphology (Fig. 3). Sequence analyses of the regions flanking the transposon inserts in the mutants that displayed semi-rough colony morphologies on TSA sucrose plates revealed 14 unique transposon mutants. Six of the transposon inserts were located in *gtf* genes encoding glycosyltransferases (Table 2), which are capable of synthesizing polymers of glucans from sucrose, and of importance for sucrose-dependent biofilm formation. Four of the isolated transposon inserts were located in the *comAB* operon (Table 2), also referred to as competence-related secretory locus (*cslAB*) (Petersen and Scheie 2000). The other transposon insertions were located in an alcohol-acetalaldehyde dehydrogenase gene, a putative oligopeptide transporter and in two different ORFs encoding putative proteins with unknown functions (Table 2).

**Table 2:** Transposon insertion sites in *S. mutans* UA159 mutants isolated in a screen for smooth or semi-rough colony morphology on TSA sucrose agar plates

Mutant designation	Transposon insertion site	Affected gene	Putative function of gene product
1B6	958790	*gtfC*	Glycosyltransferase
12H5	150205	*adhE*	Alcohol-acetalaldehyde dehydrogenase
14A8	956319	*gtfC*	Glycosyltransferase
30C6	953840	*gtfB*	Glycosyltransferase
30F6	578023	Smu_616	Hypothetical protein
36C7	959225	*gtfC*	Glycosyltransferase
41D1	272044	*comA*	CSP transport
42B6	956088	*gtfC*	Glycosyltransferase
42G11	892647	Smu_941c	Hypothetical protein
43G8	244904	*oppA*	Putative oligopeptide ABC transporter
46C7	273621	*comB*	CSP transport
47H9	271530	*comA*	CSP transport
48B3	952972	*gtfB*	Glycosyltransferase
50B1	271269	*comA*	CSP transport

## Discussion

We considered several mutagenesis strategies and transposition vectors, before we chose to employ the temperature-sensitive *repATs* origin of replication and the mariner transposon with inducible transposase. Plasmid pTV1-OK (Gutierrez et al. 1996), which served as the source of the *repATs* replicon, is a temperature-sensitive derivative of pWVO1, a broad-host-range plasmid originally isolated from *Lactococcus lactis* but with the ability to replicate in various streptococcal species. Thus, the feature that the *repATs* can initiate replication in streptococci at a permissive temperature of 30°C, but not at temperatures above 37°C is well established. Plasmid pTV1-OK is a delivery vector for Tn917 mutagenesis, however, the Tn917 transposon has been reported to have insertion hot spots in the chromosome of various bacteria, making library construction inefficient. For example, in *Streptococcus equi,* 60% of transposon inserts had occurred within a 15 kb region of the genome (Slater et al. 2003). An extreme regional preference for Tn917 mutagenesis has also been shown in *Enterococcus faecalis* and *Bacillus subtilis* (Shi et al. 2009). On the contrary, the mariner transposon system has been used in many bacterial species without any apparent insertion bias in the chromosome (Rubin et al. 1999; Bae et al. 2004; Wong and Akerley 2008; Beare et al. 2009).

Other systems than the mariner-based one have previously been used to create mutants in streptococci. A non-transposon-mediated mutagenesis strategy employed *Sau3AI* digested *S. mutans* GS-5 chromosomal DNA that was ligated to a linearized vector and used to transform *S. mutans* GS-5 (Yoshida and Kuramitsu 2002). This suicide plasmid-mediated insertion-mutagenesis procedure resulted in ∼12.000 *S. mutans* mutants, which were screened for genes that mediated sucrose independent biofilm formation. However, this procedure requires a high frequency of transformation, which is not displayed by all streptococcal strains. Cvitkovitch et al. (1998) generated a transposon mutant library using pTV1-OK, but this system is based on Tn917 and subject to extreme insertion bias as described above. Recently, ISS1 transposition mutagenesis, with a temperature-sensitive vector, has been used successfully in *S. mutans* (Thibessard et al. 2002). ISS1 appears to integrate randomly in the genome of streptococci (Thibessard et al. 2002). However, some *Streptococcus* species contain endogenous ISS1 elements which may mediate homologous recombination events between the ISS1 originating from the transposon vector and the endogenous ISS1 sequences (Thibessard et al. 2002).

We used our new mariner transposon vector to create an *S. mutans* mutant library, and performed a screen for mutants deficient in producing sucrose-dependent extracellular matrix components. *S. mutans* forms rough colonies on TSA sucrose agar plates but smooth colonies on agar plates without sucrose, and mutants that display less rough colony morphology on TSA sucrose agar plates may be impaired in the synthesis of sucrose-dependent extracellular matrix components. Six of the *S. mutans* tranposon mutants that displayed semi-rough colony morphology on TSA sucrose plates had transposon inserts in *gtf* genes encoding glycosyltransferases. Sucrose is known to be a strong inducer of *S. mutans* biofilm formation, dependent on the three glycosyltransferases GtfB, GtfC and GtfD that from sucrose synthesize different variants of glucans (Bowen and Koo 2011). Collectively, the Gtf's play a major role in the development and establishment of the extracellular matrix in sucrose-induced *S. mutans* biofilm. The *gtfB* and *gtfC* single mutants that were isolated in this study displayed semi-rough colony morphology on TSA sucrose plates, which seem to be an intermediate phenotype between the colony morphology displayed by the wild type on TSA sucrose plates and on plates without sucrose. It is possible that knockout of more than one *gtf* gene in *S. mutans* is necessary to obtain a smooth colony morphology on TSA sucrose plates (Hanada and Kuramitsu 1988). However, we found that several other genes, including the *comAB* genes (as addressed below), are also required for the rough *S. mutans* colony morphology on TSA sucrose plates. We did not isolate *gtfD* transposon mutants in this study, which is in agreement with earlier studies showing that *gtfD* mutants display growth defects (Koo et al. 2010), as during mutant library preparation we consistently avoided picking mutants which formed smaller colonies than the wild type. Four of the *S. mutans* transposon mutants that displayed a semi-rough colony morphology on TSA sucrose plates had transposon inserts in the competence-stimulating peptide (CSP) exporter genes *comAB*. ComA is an ABC transporter and ComB an accessory protein, which together constitute the secretory apparatus that is involved in processing and export of CSP. A role of CSP signaling in the production of sucrose-induced matrix components is interesting and warrants further investigations.

In our screen for *S. mutans* mutants that form semi-rough colonies on sucrose plates, we also found transposon insertions in an alcohol-acetaldehyde dehydrogenase gene, a putative oligopeptide transporter gene, and two different ORFs encoding putative proteins with unknown functions. Mutants with transposon insertion in these genes were only isolated once, unlike the case with the *gft* and *com* genes described above. For the mutants that were only isolated once, there is a risk that a secondary mutation in the genome causes the semi-rough colony morphology instead of the transposon insertion. For instance, a relatively rare event involving endogenous recombination of *gtfB* and *gtfC* in *S. mutans* can result in smooth colony morphology on sucrose plates (Narisawa et al. 2011). Therefore, the genes inactivated by transposon insertion in the four mutants that were only isolated once should only be regarded as candidates for being involved in the production of sucrose-dependent extracellular matrix components. Further analysis is required to confirm this.

After an era with an enormous development regarding gene discovery and publication of genome sequences, there is still a need of various systems to study gene function (van Opijnen and Camilli 2013). We present a new mariner transposon vector which should be a valuable tool for producing mutant libraries in streptococcal species, as it enables unbiased transposon mutagenesis. Because the transposon vector can replicate in streptococci and subsequently, after inducible transposition, can be cured by a temperature shift, it is useful for streptococcal strains that display a low plasmid transformation frequency.
